# Commencement of Berkshire Medical College

**Published:** 1865-11

**Authors:** 


### Commencement of Berkshire Medical College.

The commencement of the Berkshire Medical College occurred on Wednesday, Nov. 8th.

The exercises began at 10½ o'clock, A. M., by reading the Theses.

Prayer was offered by Rev. Dr. Todd, of Pittsfield, after which the President, Dr. H. H. Childs, briefly but impressively addressed the graduating class, and conferred the degree of Doctor of Medicine upon eighteen candidates, and the honorary degree of Doctor of Medicine upon eighteen candidates, and the honorary degree of Medicine upon Prof. Wm. C. Richards, of Pittsfield, and Robert Treat, of Wisconsin.

The exercises were concluded by a very able and exceedingly interesting valedictory, address, delivered extemporaneously by Prof. Wm. C. Richards, M. D., who was recently appointed to fill the chair of "Chemistry and Natural History," made vacant by the resignation of Prof. P. A. Chadbourne, M. D.

The following is a list of the names of the graduates and the subject of their theses:

F. S. Abbott, A. M., Mind and its Influence on Disease; Charles Bliss, Fibrin and its Uses; A. J. Brown, Pyæmia; A. S. Dian, Acute Rheumatism; J. N. Dichson, Modes of Death; G. W. Emery, Variola; Stillman Getchell, Diagnosis of Pneumonia; W. A. Jones. Hereditary Transmission; F. B. Lawson, (excused); Chas McAllister, Abscess; J. G. Page, Necessity of recognising imaginary Diseases and remedies; M. J. Powers, Puerpal Peritonitis; O. F. Searle, Syphilis; E. H. Sexton, Evidence of Utero Gestation; Arnold Stedman, Tubercular Meningitis; Hiram Temple, Asthma; John Winsor, Diagnosis.

